# Enhanced Efficiency at Maximum Power in a Fock–Darwin Model Quantum Dot Engine

**DOI:** 10.3390/e25030518

**Published:** 2023-03-17

**Authors:** Francisco J. Peña, Nathan M. Myers, Daniel Órdenes, Francisco Albarrán-Arriagada, Patricio Vargas

**Affiliations:** 1 Departamento de Física, Universidad Técnica Federico Santa María, Av. España 1680, Valparaíso 11520, Chile; 2 Millennium Nucleus in NanoBioPhysics (NNBP), Av. España 1680, Valparaíso 11520, Chile; 3 Department of Physics, Virginia Tech, Blacksburg, VA 24061, USA; 4 Departamento de Física, CEDENNA, Universidad de Santiago de Chile (USACH), Avenida Víctor Jara 3493, Estación Central 9170124, Chile; 5 Departamento de Física, CEDENNA, Universidad Técnica Federico Santa María, Av. España 1680, Valparaíso 11520, Chile

**Keywords:** magnetic cycle, quantum Otto cycle, quantum thermodynamics

## Abstract

We study the performance of an endoreversible magnetic Otto cycle with a working substance composed of a single quantum dot described using the well-known Fock–Darwin model. We find that tuning the intensity of the parabolic trap (geometrical confinement) impacts the proposed cycle’s performance, quantified by the power, work, efficiency, and parameter region where the cycle operates as an engine. We demonstrate that a parameter region exists where the efficiency at maximum output power exceeds the Curzon–Ahlborn efficiency, the efficiency at maximum power achieved by a classical working substance.

## 1. Introduction

The study of quantum thermal machines, devices such as quantum engines and refrigerators that operate on working mediums composed of quantum systems, has proved to be a fruitful research area in the last decade [1,2,3,4,5]. These efforts have been focused principally on the study of the different thermodynamic cycles, such as the Otto and the Stirling cycles, in different regimes [6,7,8,9,10] and on the use of different quantum working substances such as spins [11,12,13,14,15], quantum dots [16,17], and quantum harmonic oscillators [18,19,20,21,22]. Recent work has also examined the role that quantum properties, such as quantum coherence and quantum correlations, play in the performance of quantum thermal machines [23,24,25,26,27,28]. A primary feature of interest of quantum thermal machines is their potential to surpass the performance of their classical counterparts [29,30], which open the door to a new generation of highly efficient quantum engines and refrigerators for application in emerging quantum technologies.

While recent years have seen the experimental implementation of some quantum thermodynamic cycles [11,13,31,32], these devices serve primarily as proof-of-concept prototypes rather than a practical means of extracting useful energy in the form of work. This is since coupling a quantum thermal machine to another device to extract and use the work is a complex and challenging task within the frameworks in which these quantum engines have been implemented. A promising platform for surpassing these difficulties is quantum dots, whose easy fabrication and controllability have led to significant progress in studying their thermoelectric properties [16,33,34]. A thermodynamic cycle can be implemented with a quantum dot working medium by manipulating external parameters such as magnetic fields, resulting in an electrical current in the quantum dot during the different steps of the cycle [16,35,36,37,38]. In principle, this current can be extracted for use in another device with currently available technology.

In the context of one-electron systems and quantum dots, the famous Fock–Darwin model is very well studied. This model describes an electron in a circular semiconductor quantum dot confined by a parabolic potential under a perpendicular external magnetic field [39]. The Fock–Darwin model is fundamental to understanding the behavior of quantum dots under an external magnetic field and demonstrates the competition between geometrical confinement and the confinement induced by the external field [40]. The Fock–Darwin energy spectra for conventional quantum dots have also been verified experimentally using transport spectroscopy [40,41,42,43].

Thermodynamic endoreversibility corresponds to a nonequilibrium approximation that assumes internally reversible subsystems that then exchange energy irreversibly among themselves [44]. Thus, any dissipation arises purely from the interactions of these subsystems [45,46,47,48,49]. The common feature of endoreversible engine analysis is an additional limitation on the cycle efficiency caused by the finite rate at which heat can be exchanged between the working substance and the thermal reservoirs. By applying the endoreversible model to the Carnot cycle, Curzon and Ahlborn (CA) demonstrated that the efficiency at maximum power of the classical Carnot cycle is,

(1) $$\eta_{CA}=1-\sqrt{\frac{T_{c}}{T_{h}}},$$

where $T_{c}$ and $T_{h}$ are the cold and hot reservoirs temperatures, respectively. Notably, like the Carnot efficiency, the CA efficiency is independent of the nature of the working substance.

Recently, in [19], Deffner showed that applying the endoreversible approach to the case of an Otto cycle with a single classical or quantum harmonic oscillator as the working medium yielded an efficiency at maximum power equal to CA for the classical working medium, and an efficiency at maximum power greater than CA for the quantum working medium. One of the main conclusions of that work was that, unlike the Carnot efficiency, the Curzon–Ahlborn efficiency was not a general upper bound on the efficiency at maximum power. That is, it is entirely possible to find working substances for which the efficiency at maximum power may exceed the CA efficiency, as has been shown to be true in the case of continuous engines operating outside the linear regime [50,51]. With this in mind it is of interest to explore whether other quantum working mediums, such as quantum dots, may allow for similar boosts in engine performance.

In this work, we study a finite-time endoreversible magnetic Otto cycle, using a Fock–Darwin model quantum dot as a working substance. We find that modifying the dot geometry strongly impacts all examined cycle characteristics, including increasing the efficiency at maximum power beyond the CA efficiency. These results indicate that quantum dots can serve as a viable platform of technological interest for implementing endoreversible quantum thermodynamic cycles.

This manuscript is organized as follows. In Section 2, we introduce the model that describes our working substance, including the energy spectrum, the partition function, entropy, and internal energy. In Section 3, we describe the thermodynamic cycle under consideration, obtaining the expressions for work, heat, efficiency, and power. In Section 4, we determine the efficiency at maximum power and identify the conditions under which our system can surpass the CA efficiency. Finally, in Section 5, we provide a summary of the main conclusions of our work.

## 2. Model

Our working substance is described by the Fock–Darwin (FD) model. The FD Hamiltonian corresponds to a cylindrical quantum dot subject to an external magnetic field perpendicular to the plane in which the dot is present,

(2) $$H=\frac{1}{2m^{*}}\left[\left(p_{x}-\frac{eyB}{2}\right)+\left(p_{y}+\frac{exB}{2}\right)\right]+\frac{1}{2}m^{*}\omega_{0}^{2}\left(x^{2}+y^{2}\right),$$

where *e* is the electron charge, $m^{*}$ is the effective mass of the electron (characteristic of each material), $\omega_{0}$ is an effective parameter for the strength of the harmonic trap, and *B* is the intensity of the magnetic field in the *z* direction. The problem has cylindrical symmetry and eigenenergies,

(3) $$\begin{array}{c} E_{nm}=\hbar \Omega \left(2n+|m|+1\right)+\frac{1}{2}\hbar \omega_{c}m, \end{array}$$

where $\omega_{c}\;=\;eB/m^{*}$ is the cyclotron frequency, *n* and *m* are the radial and magnetic quantum numbers (*n* = 0, 1, 2, …and *m* = −∞, …, +*∞*), respectively, and Ω is the effective frequency of the system,

(4) $$\begin{array}{c} \Omega =\omega_{0}{\left(1+{\left(\frac{\omega_{c}}{2\omega_{0}}\right)}^{2}\right)}^{\frac{1}{2}}. \end{array}$$

Notice that when the parameter $\omega_{0}\to 0$, the energy levels of Equation (3) take the usual form of the Landau energy levels in cylindrical coordinates. For high magnetic fields, $\left(\omega_{c}/2\omega_{0}>>1\right)$, Equation (3) simplifies to,

(5) $$E_{n,m}=\frac{\hbar \omega_{c}}{2}\left(n+1/2+|m|+m\right)\;,$$

as |m|+m=0 for m<0. We note that each Landau level, labeled by *n*, is infinitely degenerate in this limit.

In this paper, we considered a low-frequency coupling for the parabolic trap given by $\omega_{0}∼2.637$ THz, which in terms of energy units corresponds to a coupling of approximately 1.7 meV. This value is comparable to the typical energy of intraband optical transitions of quantum dots [39]. The order of this transition is approximately ∼1 meV for cylindrical GaAs quantum dots with effective mass $m^{*}∼0.067m_{e}$ [39,52,53]. The quantum dot length can be calculated using $l_{dot}=\sqrt{\hbar /m^{*}\omega_{0}}∼25$ nm for the above values. The effective mass gives a cyclotron frequency of $\omega_{c}\left(B=1\right)∼2.62$ THz for an intensity of B=1 T. It is important to mention that we neglected the Zeeman splitting and the spin–orbit interaction, which is very small for GaAs systems [39,52].

The partition function for our working substance can be written as [54],

(6) $$Z_{d}=\frac{\omega_{+}\omega_{-}}{4\omega_{0}^{2}}\csc h\left(\frac{\hbar \beta \omega_{+}}{2}\right)\csc h\left(\frac{\hbar \beta \omega_{-}}{2}\right),$$

where $\beta =1/k_{B}T$ is the inverse temperature and the frequencies $\omega_{\pm }$ are given by,

(7) $$\omega_{\pm }^{2}=\frac{1}{2}\left(\omega_{c}^{2}+2\omega_{0}^{2}\pm \omega_{c}\sqrt{\omega_{c}^{2}+\omega_{0}^{2}}\right).$$

Note that if $\omega_{0}\to 0$, then $\omega_{-}\to 0$ and $\omega_{+}\to \omega_{c}$, recovering the typical partition function for the Landau problem.

The entropy, S(T,B), and internal energy, U(T,B), are derived from the partition function using,

(8) $$S\left(T,B\right)=k_{B}\ln Z_{d}+k_{B}T{\left(\frac{\partial \ln Z_{d}}{\partial T}\right)}_{B},$$

and,

(9) $$U\left(T,B\right)=k_{B}T^{2}{\left(\frac{\partial \ln Z_{d}}{\partial T}\right)}_{B}.$$

In particular, the internal energy is expressed entirely in terms of β, $\omega_{+}$, and $\omega_{-}$,

(10) $$U\left(T,B\right)=\frac{1}{2}\left[\hbar \omega_{+}\coth\left(\frac{\hbar \beta \omega_{+}}{2}\right)+\hbar \omega_{-}\coth\left(\frac{\hbar \beta \omega_{-}}{2}\right)\right].$$

Note that this expression is simply the sum of the internal energies of two oscillators, one with frequency $\omega_{+}$ and the other with frequency $\omega_{-}$. Conversely, the entropy cannot be decomposed in this manner due to the prefactor present in the partition function in Equation (6). This prefactor accounts for the degeneracy of the energy levels and depends on the external magnetic field. Only in the absence of this term can the entropy be expressed as the sum of the entropies of two independent oscillators with frequencies $\omega_{+}$ and $\omega_{-}$.

**Figure 1:**
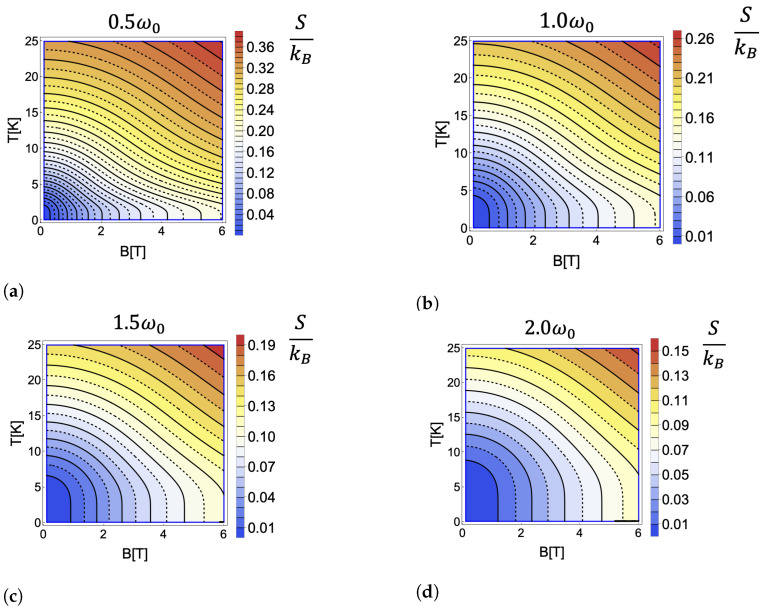
(**a**–**d**) Contour maps showing constant entropy curves as a function of temperature, *T*, (in Kelvin) and the external magnetic field *B* (in Tesla) for different values of the geometric confinement with $\omega_{0}=2.67$ THz.

**Figure 2:**
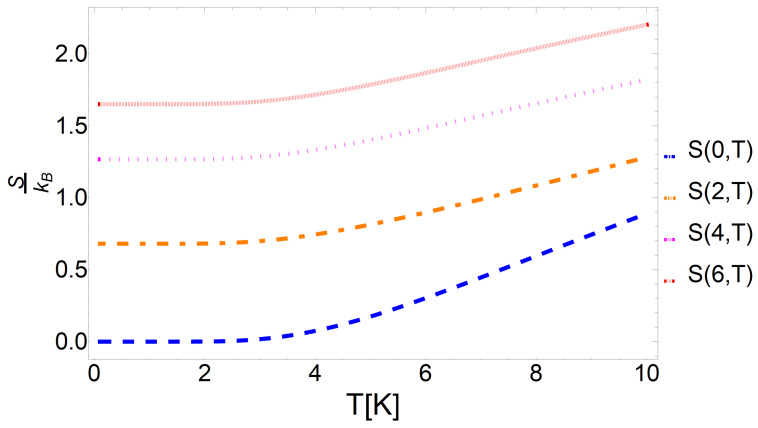
Entropy as a function of temperature for magnetic field strengths of B=0 T (blue), B=2 T (orange), B=4 T (magenta), and B=6 T (red).

While these thermodynamic quantities are widely discussed in the literature, here, we pay special attention to the conditions under which the entropy remains constant, as this will prove a key aspect of our analysis of the cycle performance. With this in mind, we seek a relationship between the temperature and the external magnetic field that holds the entropy constant. In Figure 1, we plotted the isentropic trajectories as a function of the temperature and magnetic field. From this plot, we see that the temperature and magnetic field are inversely related, i.e., when we increase the magnetic field, the temperature must decrease if we wish to hold the entropy constant. In Figure 2, we plotted the entropy as a function of temperature for a range of magnetic field strengths. From this plot, we observe that, at low temperatures, there is a large region where the entropy remains almost constant. In this region, the entropy depends only on the partition function degeneracy term, which is proportional to the value of the external field. Thus, to observe changes in entropy, a considerable increase in the temperature is required. This results in the near-vertical isentropic lines observed at low temperatures in Figure 1.

## 3. The Endoreversible Otto Cycle

**Figure 3:**
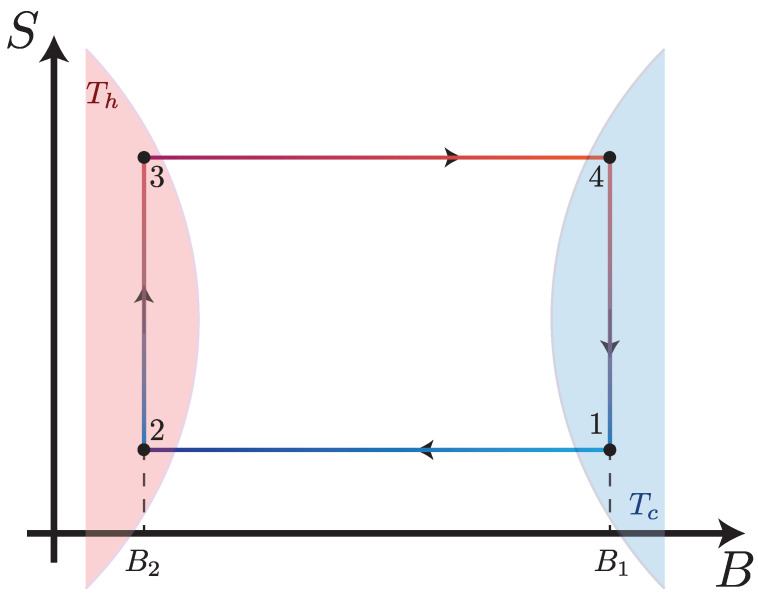
Entropy versus external field diagram for the endoreversible Otto Cycle. Note that the system is only in contact with the thermal reservoirs during the isochoric (vertical) strokes. Under the assumptions of endoreversibility, the working substance does not fully thermalize to the temperatures of the hot and cold reservoirs, $T_{h}$ and $T_{c}$, at points 3 and 1.

The Otto cycle sketched on an entropy–magnetic field diagram in Figure 3, consists of four strokes: two isentropic (horizontal lines) processes and two isochoric processes (vertical lines). During the first process (1→2), the working substance is disconnected from the thermal reservoir, and the external magnetic field is changed adiabatically from $B_{1}$ to $B_{2}$. Since $B_{1}<B_{2}$ the internal energy of the working medium increases during this process, despite the fact that the working medium temperature decreases. Thus, this stroke is properly classified as an isentropic compression in which the magnetic field plays a role analogous to the inverse of the volume. The next process (2→3) is an isochoric heating stroke during which the working substance is put in contact with a thermal reservoir at temperature $T_{h}$ and allowed to exchange heat while the magnetic field remains constant. The next stroke (3→4) is accomplished by disconnecting the working substance from the thermal reservoir and changing the magnetic field adiabatically from $B_{2}$ back to $B_{1}$. As the internal energy of the working medium decreases during this stroke, despite increasing in temperature, it corresponds to an isentropic expansion. Finally, the last process (4→1) is an isochoric cooling stroke, during which the working substance is put in thermal contact with a reservoir at temperature $T_{c}<T_{h}$ and allowed to exchange heat while the magnetic field is again held constant. We note that the inverted behavior of the temperature during the compression and expansion strokes (in comparison to the typical Otto cycle) arises due to the inverse relationship between the temperature and magnetic field necessary to maintain a constant entropy, as observed in the negative slope of the isentropic curves in Figure 1.

The fact that the temperature decreases during the isentropic compression stroke can have negative impacts on the cycle performance, especially in the parameter regions where the decrease is significant, such as the low-temperature, low-magnetic field regimes seen in Figure 1. In these regimes the isentropic curves become nearly vertical in temperature, indicating that a small increase in the magnetic field must correspond to a large decrease in the temperature if the compression stroke is to remain isentropic. The low temperature of the working medium will then result in a large amount of heat being absorbed from the hot reservoir during the subsequent heating stroke. Since the efficiency is measured by the ratio of total work to heat absorbed from the hot reservoir, a cycle implemented in this region will have a significantly reduced efficiency.

Thermodynamically, the cycle is characterized by the temperatures of the two thermal reservoirs ($T_{h}$ and $T_{c}$) and by the initial and final values of the magnetic field ($B_{1}$ and $B_{2}$). Under the assumption of endoreversibility, we note that the working medium is assumed to always be in a state of local equilibrium with a well-defined temperature but that it never fully thermalizes with the reservoirs. Following the analysis established in [19], we determine the heat exchanged with the reservoirs during the isochoric heating stroke (2→3) using,

(11) $$Q_{\mathrm{in}}=U_{3}\left(T_{3},B_{2}\right)-U_{2}\left(T_{2},B_{2}\right),$$

where we note $T_{3},T_{2}\neq T_{h}$. The temperatures $T_{2}$ and $T_{3}$ satisfy the following conditions,

(12) $$T\left(0\right)=T_{2}\;\mathrm{and}\;T\left(\tau_{h}\right)=T_{3}\;\mathrm{with}\;T_{2}<T_{3}\leq T_{h},$$

where $\tau_{h}$ is the duration of the heating stroke. We can explicitly model the temperature change from $T_{2}$ to $T_{3}$ by applying Fourier’s law of heat conduction,

(13) $$\frac{dT}{dt}=-\alpha_{h}\left(T\left(t\right)-T_{h}\right),$$

where $\alpha_{h}$ is a constant that depends on the working medium’s thermal conductivity and heat capacity. Solving Equation (13) results in

(14) $$T_{3}-T_{h}=\left(T_{2}-T_{h}\right)e^{-\alpha_{h}\tau_{h}}.$$

The isentropic expansion stroke (from 3→4) is performed identically to the case of a quasi-static cycle. Since the working medium is decoupled from the thermal reservoirs during this stroke, the work is determined entirely from the change in internal energy,

(15) $$W_{\exp}=U_{4}\left(T_{4},B_{1}\right)-U_{3}\left(T_{3},B_{2}\right).$$

The isochoric cooling stroke (4→1) can be modeled identically to the heating stroke. The heat exchanged with the cold reservoir is

(16) $$Q_{\mathrm{out}}=U_{1}\left(T_{1},B_{1}\right)-U_{4}\left(T_{4},B_{1}\right),$$

where $T_{1}$ and $T_{4}$ satisfy the conditions

(17) $$T\left(0\right)=T_{4}\;\mathrm{and}\;T\left(\tau_{c}\right)=T_{1}\;\mathrm{with}\;T_{4}>T_{1}\geq T_{c}.$$

As in the heating stroke, the temperature change can be modeled by Fourier’s law,

(18) $$\frac{dT}{dt}=-\alpha_{c}\left(T\left(t\right)-T_{c}\right),$$

By solving Equation (18), we obtain

(19) $$T_{1}-T_{c}=\left(T_{4}-T_{c}\right)e^{-\alpha_{l}\tau_{c}}.$$

Finally, the work done during the adiabatic compression stroke (1→2) can be found from the change in internal energy,

(20) $$W_{\mathrm{comp}}=U_{2}\left(T_{2},B_{2}\right)-U_{1}\left(T_{1},B_{1}\right).$$

The efficiency of the engine can then be found from the ratio of the total work and the heat exchanged with the hot reservoir,

(21) $$\eta =-\frac{W_{\mathrm{comp}}+W_{\exp}}{Q_{\mathrm{in}}}.$$

The power output is given by the ratio of the total work to the cycle duration,

(22) $$P=-\frac{W_{\mathrm{comp}}+W_{\exp}}{\gamma (\tau_{h}+\tau_{c})},$$

where γ>1 is a multiplicative factor that incorporates the duration of the isentropic strokes [19].

As the entropy remains constant during the isentropic process, we can obtain a relationship between *T* and *B* from the condition dS(T,B)=0. This first-order differential equation is given by,

(23) $$\frac{dB}{dT}=-\frac{{\left(\frac{\partial S}{\partial T}\right)}_{B}}{{\left(\frac{\partial S}{\partial B}\right)}_{T}}.$$

For the case of a Fock–Darwin model quantum dot, even though the analytic form of the partition function is known, as given in Equation (6), this equation does not yield a straightforward analytical solution. Nevertheless, it can be solved numerically, as was done to determine the constant entropy curves in Figure 1. In addition, it is useful to define the “magnetic length” as

(24) $$l_{B}=\sqrt{\frac{\hbar }{eB}}.$$

By taking the ratio of the magnetic lengths,

(25) $$r=\frac{l_{B_{2}}}{l_{B_{1}}}=\sqrt{\frac{B_{1}}{B_{2}}},$$

we obtain a quantity analogous to the compression ratio for the classical Otto cycle.

In the limit of $\tau_{h},\tau_{c}\to \infty$, the working medium will fully thermalize and we expect to recover the quasi-static cycle behavior. We see that in this limit,

(26) $$\begin{array}{c} \lim_{\tau_{h}\to \infty }\left(T_{3}-T_{h}=\left(T_{2}-T_{h}\right)e^{-\alpha_{h}\tau_{h}}\right)\to T_{3}=T_{h},\\ \lim_{\tau_{c}\to \infty }\left(T_{1}-T_{c}=\left(T_{4}-T_{c}\right)e^{-\alpha_{c}\tau_{c}}\right)\to T_{1}=T_{c}. \end{array}$$

However, this limit also results in a vanishing power output, as seen from Equation (22).

## 4. Results and Discussions

**Figure 4:**
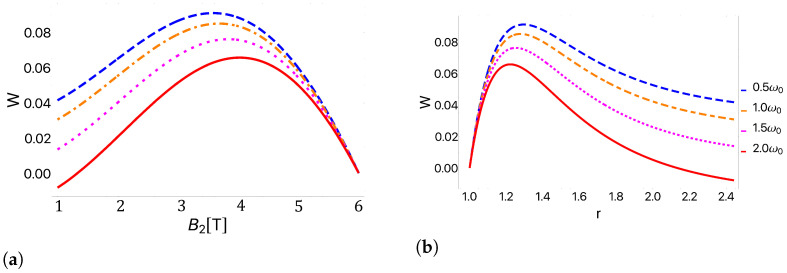
Total work as a function of (**a**) the external field and (**b**) the compression ratio for geometric confinement frequencies of $0.5\omega_{0}$ (blue), $\omega_{0}$ (orange), $1.5\omega_{0}$ (magenta), and $2\omega_{0}$ (red). We used $T_{c}=13$ K, $T_{h}=25$ K, and $B_{1}=1$ T. Note the clear decrease in the net work with increasing values of the dot confinement. In particular, for these values a transition to negative net work is observed for the case of $2\omega_{0}$ (red curve). At this transition point the cycle switches from behaving as an engine to behaving as a refrigerator.

**Figure 5:**
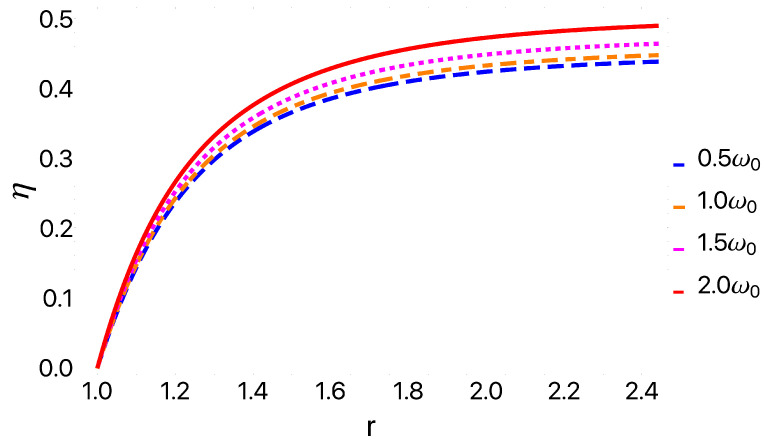
Efficiency as a function of *r* for geometric confinement frequencies of $0.5\omega_{0}$ (blue), $\omega_{0}$ (orange), $1.5\omega_{0}$ (magenta), and $2\omega_{0}$ (red). We used $T_{c}=13$ K and $T_{h}=25$ K. We observe that the system’s efficiency increases as we increase the geometric confinement.

We began by considering a specific example with bath temperatures of $T_{c}=13$ K and $T_{h}=25$ K in order to illustrate the cycle performance. These results are presented in Figure 4, for the work extracted and in Figure 5 for the efficiency. In Figure 4, we observe that as the parabolic trap parameter $\omega_{0}$ decreases, the amount of work extracted increases. Conversely, the efficiency presented in Figure 5 follows the opposite behavior, growing as $\omega_{0}$ increases. This comparison demonstrates a clear trade-off between work and efficiency. In addition, we observe in Figure 4 that there exists a region in which the total work becomes negative for the case of $2\omega_{0}$, indicating that our cycle is no longer behaving as an engine in this regime. With this in mind, we next turned to explore the parameter regimes where the cycle functioned as different types of thermal machines.

In general, there exist four possible types of thermal machines, corresponding to all possible combinations of heat and work flow consistent with the first and second laws of thermodynamics. An engine corresponds to a positive work output, along with a heat flow from the hot bath into the working medium and from the working medium into the cold bath. A refrigerator corresponds to a negative work output, along with a heat flow from the cold bath into the working medium and from the working medium into the hot bath. A heater corresponds to a negative work output and a heat flow from the working medium into both baths. Finally, an accelerator corresponds to a negative work output along with a heat flow from the hot bath into the working medium and from the working medium into the cold bath. We note that the heater and accelerator regimes generally emerge due to nonequilibrium behavior, and thus we do not expect a cycle operating endoreversibly to act as either of these types of thermal machines.

By numerical calculation, we determined the results of Equations (11), (15), (16), and (20) across the parameter space. By comparing the signs of the resulting heat and work flows, we determined the regions where the cycle behaved as an engine, refrigerator, heater, or accelerator.

**Figure 6:**
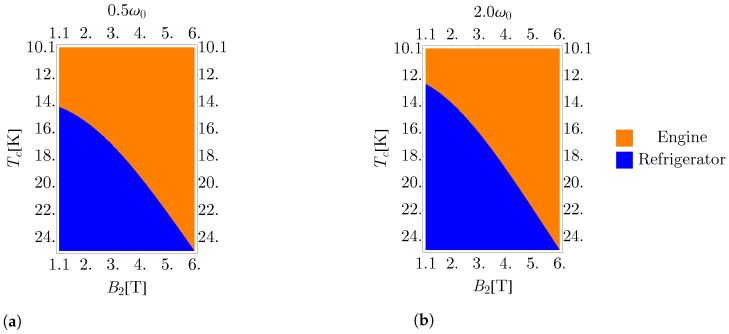
Cycle behavior as a function of the external magnetic field strength and cold bath temperature for a geometric confinement of (**a**) 0.5$\omega_{0}$ and (**b**) $2\omega_{0}$. Note the increased size of the refrigerator region as the value of the parabolic trap frequency increases.

Figure 6 shows the regions where the cycle operates as an engine or as a refrigerator as a function of the cold bath temperature and the external magnetic field for two different values of $\omega_{0}$ while holding the temperature of the hot reservoir constant at $T_{h}=25$ K. We observe that by increasing the frequency of geometric confinement, the region in which the cycle operates as an engine is reduced. This behavior can be understood from Figure 4, where it is observed that as the trap frequency increases, the extracted work decreases, resulting in a transition into the regime of negative work. From Figure 6, we see that increasing the value of $T_{c}$ increases the value of the magnetic field at which the transition to the refrigerator regime occurs. Recalling Figure 4b, increasing $T_{c}$ corresponds to shifting the peak to the left, and thus also shifting to the left the critical point at which the transition to the negative work regime occurs while maintaining the same qualitative behavior.

**Figure 7:**
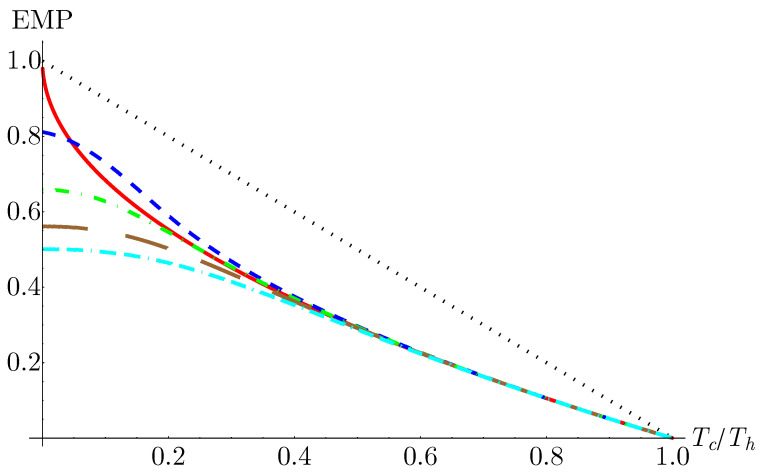
Efficiency at maximum power as a function of the bath temperature ratio for $0.5\omega_{0}$ (blue, short dashed), $1.0\omega_{0}$ (green, dot-dashed), $1.5\omega_{0}$ (brown, long dashed), and $2.0\omega_{0}=$ (cyan, dot-dash-dashed). The Carnot (black, dotted) and Curzon-Ahlborn (red, solid) efficiencies are provided for comparison. We observe that the efficiency at maximum power exceeds CA for lower values of dot confinement frequency at low bath temperature ratios. Parameters are $T_{h}$ = 25 K and magnetic field $B_{h}=$ 12 T.

To numerically determine the EMP, we fixed the temperature of the hot bath at $T_{h}=25$ K and the high magnetic field value at $B_{h}=$ 12 T. We then varied the cold bath temperature across the range $T_{c}\in \left[0\;K,25\;K\right]$ and the low magnetic field value from $B_{l}\in \left[0\;T,12\;T\right]$. This range of parameter space was selected to ensure that the peak in the work as a function of the compression ratio (see Figure 4b) was captured for all combinations of bath temperatures. For each bath temperature ratio, we scanned the parameter space to determine the magnetic field strengths that produced the maximum power output and then determined the efficiency at that maximum power. In Figure 7, we plotted the EMP as a function of the bath temperature ratio $T_{c}/T_{h}$ for different values of $\omega_{0}$. These EMPs were then compared with the CA efficiency, found in Equation (1).

From Figure 7, we can see that the geometric confinement frequency has a significant impact on the EMP at low bath temperature ratios. Lower values of the confinement frequency yield higher EMPs. In particular, for the case of $\omega_{0}=0.5$, we see that there exists a range of bath temperatures where the EMP exceeds the CA efficiency. At higher bath temperatures, we see the EMP converges towards the CA efficiency for all values of the confinement frequency, as is expected in the high-temperature, classical limit.

Previous results have shown that the EMP of an endoreversible quantum Otto engine with a pure harmonic oscillator as the working medium exceeds the CA efficiency [19]. As the partition function in Equation (6) simplifies to that of a two-dimensional isotropic harmonic oscillator in the limit $\omega_{0}\to 0$ [54], finding that a low $\omega_{0}$ leads to a region where the CA efficiency can be exceed is consistent. Here, we further demonstrated that the addition of a geometric confinement had a strictly detrimental impact on the engine performance.

An additional feature of note is the asymptotic flattening of the EMP as the bath temperature ratio approaches zero. This behavior again arises from the structure of the isentropic curves seen in Figure 1. As the entropy is nearly independent of temperature in the low temperature regime, the value of the compression ratio that maximizes the power output (and thus the value of the EMP) does not change significantly until the system moves out of this regime. This explains why the asymptotic behavior is especially pronounced at high values of the confinement frequency, which display larger ranges of temperatures under which the isentropic curves are nearly vertical with respect to the magnetic field.

## 5. Conclusions

In this work, we analyzed the performance of an endoreversible Otto cycle using a quantum-dot-trapped electron in the presence of an external magnetic field as a working substance. Modeling our working substance using the Fock–Darwin Hamiltonian, we found that the net work, power, and efficiency depended strongly on the intensity of the parabolic trapping frequency, as well as influencing whether the cycle behaved as either an engine or a refrigerator. In particular, we found that larger values of the trapping frequency led to an increased efficiency but reduced work, power, and EMP. Despite this, we demonstrated that there existed a parameter regime in which the cycle EMP exceeded the Curzon–Ahlborn efficiency for small values of the trapping frequency. Furthermore, we found that the strongly nonlinear temperature dependence of the entropy for the Fock–Darwin model quantum dot had distinct signatures in the engine performance, namely an asymptotic behavior of the EMP at low temperatures.
